# DNA Repair and Replication-Related Gene Signature Based on Tumor Mutation Burden Reveals Prognostic and Immunotherapy Response in Gastric Cancer

**DOI:** 10.1155/2022/6469523

**Published:** 2022-01-11

**Authors:** Lei Zhang, Dahai Hu, Shuchen Huangfu, Jiaxin Zhou, Wei Wang, Shijin Liu, Hui Tang, Jinghua Pan, Yunlong Pan

**Affiliations:** ^1^Department of Gastrointestinal Surgery, The First Affiliated Hospital of Jinan University, Guangzhou 510632, China; ^2^Department of Oncology Surgery, The Second Affiliated Hospital of Bengbu Medical College, Bengbu, Anhui Province 233080, China; ^3^Clinical Medicine Research Institute, The First Affiliated Hospital of Jinan University, Guangzhou 510632, China; ^4^International School, Jinan University, Guangzhou, Guangdong 510632, China; ^5^Jiangmen Maternity and Child Health Care Hospital, Huizhou 52900, China

## Abstract

The genomic variant features (mutations, deletions, structural variants, etc.) within gastric cancer impact its evolution and immunogenicity. The tumor has developed several coping strategies to respond to these changes by DNA repair and replication (DRR). However, the intrinsic relationship between the associated DRR-related genes and gastric cancer progression remained unknown. This study selected DRR-related genes with tumor mutation burden based on the TCGA (The Cancer Genome Atlas) database of gastric cancer transcriptome and mutation data. The prognosis model of seven genes (*LAMA2*, *CREB3L3*, *SELP*, *ABCC9*, *CYP1B1*, *CDH2*, and *GAMT*) was constructed by a univariate and LASSO regression analysis and divided into high-risk and low-risk groups with the median risk score. Survival analysis showed that overall survival (OS) was lower in the high-risk group than that in the low-risk group. Moreover, patients with gastric cancer in the high-risk group have worse survival in different subgroups, including age, gender, histological grade, and TNM stage. The nomogram that included risk scores for DRR-related genes could accurately foresee OS of patients with gastric cancer. Interestingly, the tumor mutation burden score was higher in the low-risk group than that in the high-risk group, and the risk score for DRR-related genes was negatively correlated with tumor mutation burden in gastric cancer. Next, we further combined the risk score and tumor mutation burden to evaluate the prognosis of gastric cancer patients. The low-risk cohort had a better prognosis than the high-risk cohort in the high tumor mutation burden subgroup. The number of mutation types in the high-risk group was lower than that in the low-risk group. In the immune microenvironment of gastric cancer, more naïve B cells, memory resting CD4+ T cells, Treg cells, monocytes cells, and resting mast cells were infiltrated in the high-risk group. At last, PD-L1 and IAP expressions were negatively correlated with the risk scores; patients with gastric cancer in the low-risk group showed better immunotherapy outcomes than those in the high-risk group. Overall, the DRR-related gene signature based on tumor mutation burden is a novel biomarker for prognostic and immunotherapy response in patients with gastric cancer.

## 1. Introduction

Gastric cancer (GC) is one of the most common malignant tumors worldwide and the second leading cause of cancer-related death [1, 2]. Its high incidence, high mortality, and poor prognosis pose a severe threat to human health and life. At present, surgical resection is the leading choice for the treatment of patients with early GC, and chemotherapy is the essential treatment for patients who cannot be resected or have advanced metastasis [3, 4]. However, GC is highly heterogeneous in biology and genes, resulting in less optimal surgical resection and chemotherapy results [5]. Therefore, there is an urgent need to explore more effective treatment strategies.

Tumor mutational burden (TMB) is defined as the total number of somatic gene coding errors, base substitutions, insertions, or deletions detected per million bases [6, 7]. TMB is a quantitative biomarker that reflects the total number of mutations carried by tumor cells, and tumor cells with high TMB will have higher levels of neoantigens [8]. It is thought to assist the immune system in recognizing tumors and stimulating the proliferation of antitumor T cells [9]. Both studies reported that TMB in GC was associated with OS and clinical benefit rate, and high TMB can be used as a biomarker for the clinical efficacy of immune checkpoint blocker (ICB) in GC patients [6, 10].

Defects in replication repair-associated DNA polymerases often manifest an ultrahigh TMB. DNA repair and replication (DRR) is an essential pathway for cells to cope with DNA damage [11, 12]. Recent studies have shown that increasing DNA damage and decreasing the DNA repair capacity of cancer cells lead to genomic distortion [13, 14]. Ying J et al. found that BRCA2, ATM, MSH6, and ATR exhibited high-frequency mutations in the DRR pathway, and TMB-high polymerase unknown significance variants were closely associated with DRR pathway genes and polymerase mutation features and prolonged OS, suggesting an essential role of DRR-related gene detection in cancer prognosis [15]. In addition, DRR-related genes are highly correlated with tumor chemotherapy resistance [16]. A recent clinical trial showed that cancer patients with BRCA1/2 mutations had higher response rates when treated with poly-ADP-ribose polymerase (PARP) inhibitors [17]. Moreover, numerous studies have shown that tumors with DRR mutations are more sensitive to platinum-based therapies. DRR-related genes may provide potential biomarkers for clinical prognosis and immunotherapy in GC. Combining the PARP inhibitor olaparib with the dual WEE1/PLK1 inhibitor AZD1775 to increases the effects of olaparib on GC cell growth inhibition and induction of apoptosis by blocking the DNA damage repair pathway [18]. Taken together, DRR-related genes may provide potential biomarkers for clinical prognosis and immunotherapy in GC.

To identify a novel biomarker for prognosis and therapeutic response in GC based on DRR-related genes, we first screened DRR-related genes in GC based on tumor mutation burden and constructed prognostic models. Then, we comprehensively evaluated the DRR-related gene signature that could predict the prognosis of GC patients and analyzed in detail the relationship between the DRR-related gene signature and the immune microenvironment in GC. Our study identified seven DRR-related genes as tumor signatures, with high sensitivity for GC's prognostic and immunotherapeutic response.

## 2. Materials and Methods

### 2.1. Patients and Clinical Specimens

RNA sequencing (RNA-seq) and matching complete clinical information (age, gender, histological grade, survival status, and stage) of GC (*n* = 407) were retrieved from the Cancer Genome Atlas (TCGA, https://portal.gdc.cancer.gov) on July 5, 2021. Fragments with a million per thousand base (FPKM) value are normalized to transcripts per thousand base million (TPM).

### 2.2. Identification of DRR-Related DEGs and Venn Graph

The limma package in R V4.0.5 (https://www.r-project.org; |log2 fold change |>1, FDR <0.05) analyzes DEGs, the volcano maps for differential genes are utilized the ggpolt2 package in R software. The Venn diagram of the intersecting genes of DEGs and TMBs uses the Venn package.

### 2.3. Univariate Cox Analysis and Construction of the Prognostic Model

Using DRR differential genes data, the survival package is used for univariate Cox regression analysis. The least absolute shrinkage and selection operator (LASSO) regression algorithm for feature selection, using 10-fold cross-validation, the above analysis uses the R software package glmnet. For Kaplan–Meier curves, *p*-value and hazard ratio (HR) with 95% confidence interval (CI) were generated by log-rank tests and univariate Cox proportional-hazards regression. All analytical methods above and R packages were performed using R software version 4.0.5 (The R Foundation for Statistical Computing, 2021). *p* < 0.05 was considered statistically significant.

### 2.4. Construction of the Nomogram Graph Based on the Prognostic Model

The “rms” package in R builds a nomogram based on OS with independent prognostic factors. Use the AUC value to test the ability of the nomogram to distinguish survival. Construct a calibration curve of the nomogram to test the 1-, 3-, and 5-year survival probabilities based on the nomogram and actual observations.

### 2.5. Estimation of Stromal and Immune Cells in Malignant Tumor Tissues Using Expression Data

The ESTIMATE algorithm-generated matrix and immune scores are used to estimate the level of infiltrating matrix and immune cells in GC tissue and tumor purity through expression profiles. Then, we used the Wilcoxon rank-sum test to compare the differences in tumor purity, stroma, and immune scores between the high- and low-risk groups. Deconvolution results for the tumor-infiltrating immune component were yielded with data gleaned from the TCGA database, which is analyzed by the CIBERSORT algorithm.

### 2.6. Calculation of TMB Scores and Somatic Mutation Analysis

TMB is defined as the total number of somatic gene coding errors, base substitution, insertion, or deletion detected per million bases. Perl script was used to calculate the mutation frequency of the number of variations/exon length of each sample. The “Maftools” package calculated the somatic mutations in different GCs and the mutation distribution was mapped using the ggplot2 package.

### 2.7. Statistical Analysis

Statistical analysis is performed by R (version 4.0.5). The Wilcoxon rank-sum test presents comparisons between the two groups, while the Kruskal–Wallis test assesses multiple comparisons. The survminer package determines the demarcation point of each subgroup in R. The Kaplan–Meier curve of OS analysis was presented between different subgroups, and then the log-rank test was performed. Multivariate Cox regression analysis is used to evaluate the association between OS and clinicopathological characteristics and risk scores. The forestplot package visualizes these in R. AUC depicts the 1-, 3-, and 5-year survival rates and is used to assess the predictive power of risk score. Bonferroni's test corrects the *p*-value. *p* < 0.05 on both sides was considered statistically significant.

## 3. Results

### 3.1. Identification of DNA Repair and Replication-Related Prognostic Genes in High and Low TMB GC Groups

**Figure 1:**
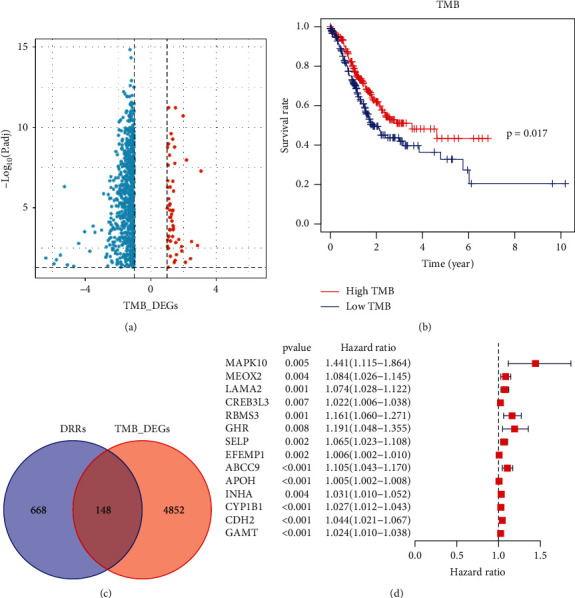
Identification of DRR-related prognostic genes in gastric cancer. (a) Volcano map of TMB differential genes. (b) Survival analysis of high and low TMB. (c) Venn diagram of DRR-related genes and TMB differential genes. (d) Univariate Cox regression analysis of intersecting genes.

Firstly, GC mutation data were downloaded from TCGA, and 816 differential genes were identified according to the high and low TMB GC groups (Figure 1(a), |log2FC| > 1, *p* < 0.05)). Moreover, the high TMB group in GC has better survival (Figure 1(b), *p* < 0.05). A total of 10,315 genes were identified by entering the search term “DNA repair and replication” from GeneCards (https://www.genecards.org), and the top 5000 genes were selected. The two groups of genes were intersected, and 148 genes were overlapped (Figure 1(c)). Univariate Cox regression analysis screened 14 genes (*MAPK10*, *MEOX2*, *LAMA2*, *CREB3L3*, *RBMS3*, *GHR*, *SELP*, *EFEMP1*, *ABCC9*, *APOH*, *INHA*, *CYP1B1*, *CDH2*, and *GAMT*) that were associated with GC prognosis (Figure 1(d), *p* < 0.05).

### 3.2. Risk Score for DRR-Related Gene Correlated with Prognosis of GC Patients

**Figure 2:**
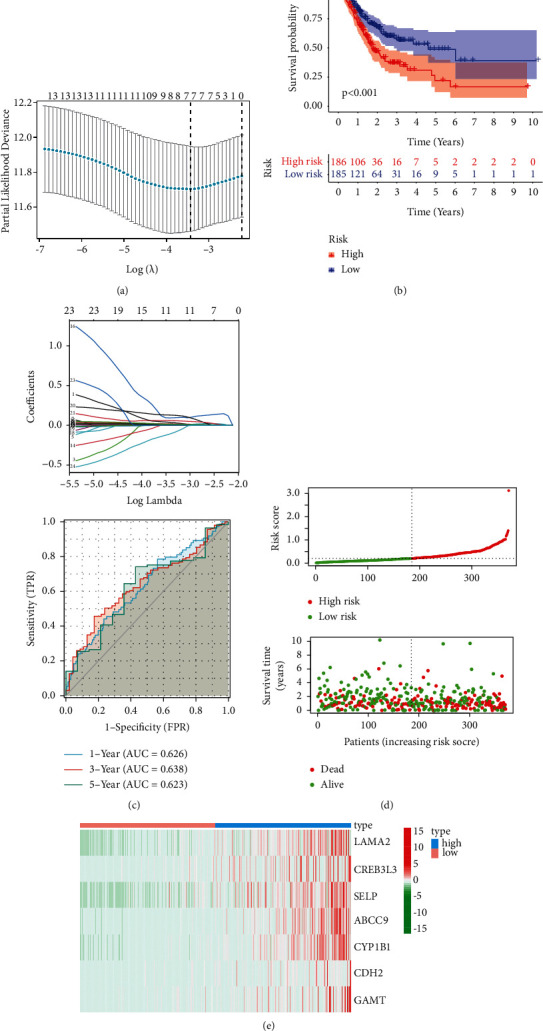
Risk score for DRR-related gene correlated with prognosis of GC patients. (a) LASSO analysis revealing the minimal lambda. (b) Survival status and risk score. (c) Time-dependent ROC curve. (d) Survival curve illustrating the overall survival of the GC patients. (e) Heatmap visualizing the expression pattern of the seven-candidate DRR-related genes.

Next, we constructed a risk score of DRR-related genes in GC. LASSO regression prognostic model was constructed from 14 genes screened by univariate Cox regression, and finally, a total of seven genes (*LAMA2*, *CREB3L3*, *SELP*, *ABCC9*, *CYP1B1*, *CDH2*, and *GAMT*) were constructed in this risk score (Figure 2(a)). The best performance of the risk score was achieved using these seven genes. The model function was as follows: risk score = (0.013918321 × *LAMA2* expression level) + (0.008279412 × *CREB3L3* expression level) + (0.71002582 × *RMI2* expression level) + (0.00495859 × *SELP* expression level) + (0.022154282 × *ABCC9* expression level) + (0.010346169 × *CYP1B1* expression level) + (0.01145852 × *GAMT* expression level). In total, 186 of the 371 GC samples were classified as a high-risk group, and the remaining 185 were classified as a low-risk group according to the median risk score. Survival analysis showed that overall survival (OS) was lower in the high-risk group than that in the low-risk group (Figure 2(b), *p* < 0.05). Receiver operating characteristic (ROC) curves verified AUC of 0.626, 0.638, and 0.623 at 1, 3, and 5 years, respectively (Figure 2(c)). The risk curves showed a positive correlation between prognostic model scores and patient risk values, and those low-risk patients had a higher survival rate than high-risk patients (Figure 2(d)). Heatmap visualizing the gene expression patterns used in the risk model showed that all seven genes in the prognostic model were highly expressed in the high-risk group (Figure 2(e)).

### 3.3. Construction and Verification of a DRR-Related Prognostic Model in GC

**Figure 3:**
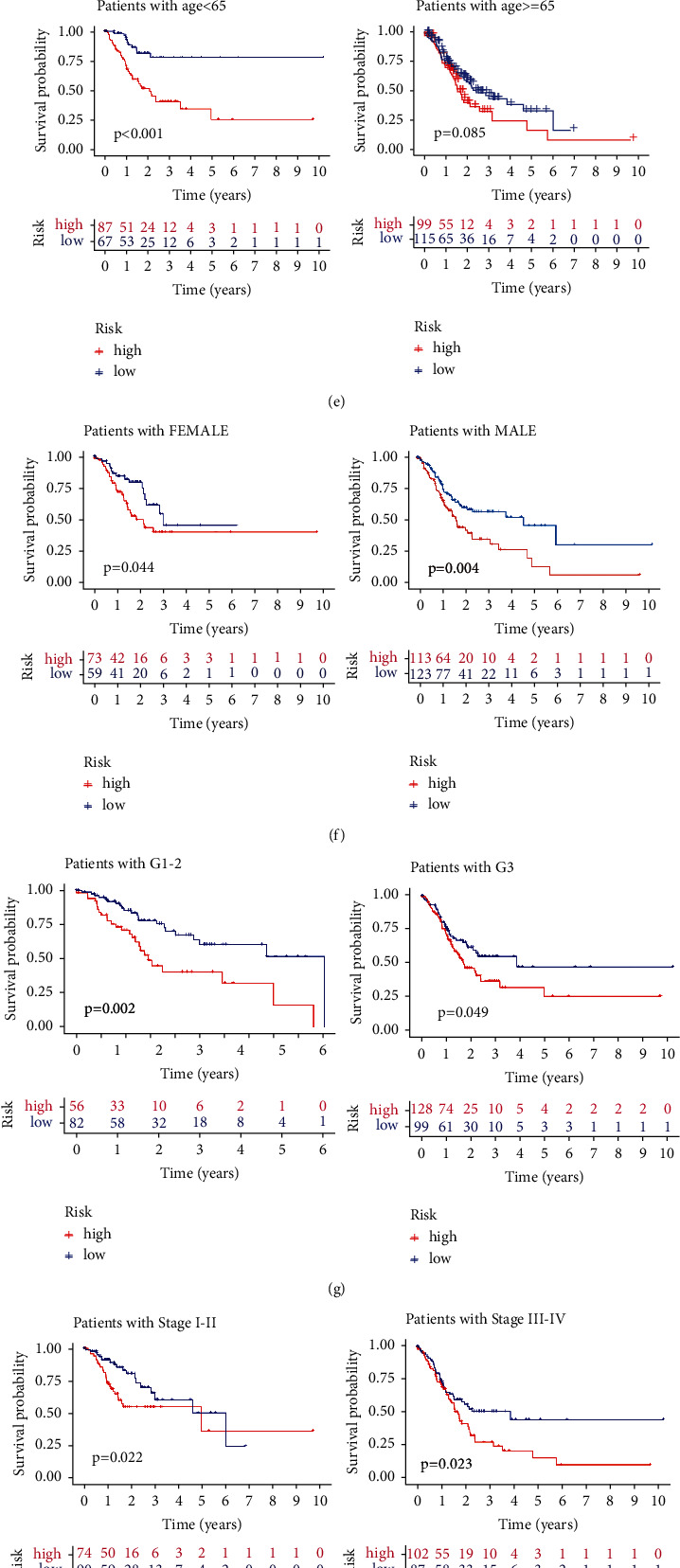
The correlation of risk score with gastric patients' clinicopathological characteristics. (a–d) The distribution of risk scores in the different ages, genders, histologic grades, and TNM stages. (e–h) The risk score could predict the survival of patients with different ages, genders, histologic grades, and TNM stages.

**Figure 4:**
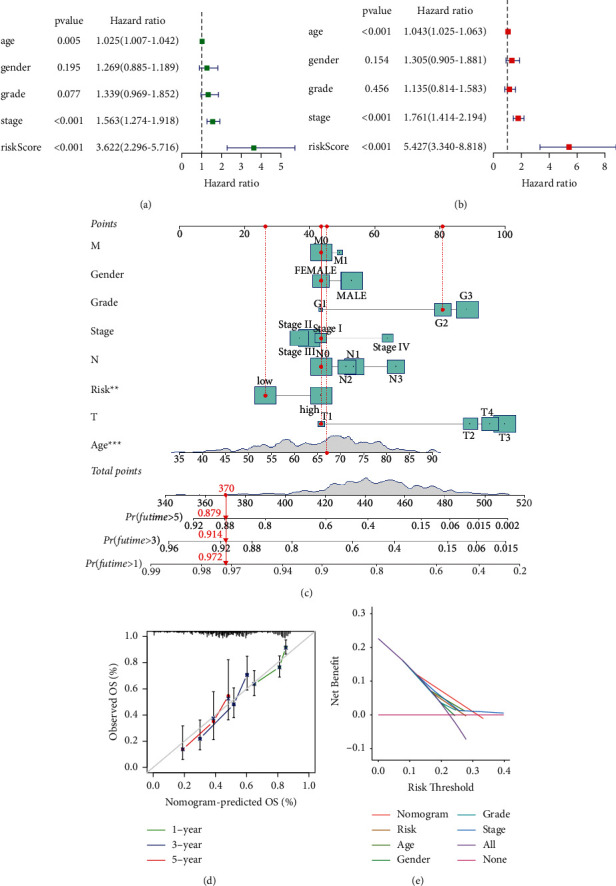
Construction and verification of a DRR-related prognostic model in gastric cancer. (a) Univariate analysis on the risk score. (b) Multivariate Cox analysis on the risk score. (c) Nomogram based on the prognosis associated DRR-related genes. (d) The calibration curves comparing the estimated 1-, 3-, and 5-year survival probability with the actual survival probability of GC patients. (e) DCA of clinical features and risk model.

Moreover, we evaluated the prognostic value of risk score for DRR-related genes in different subgroups of GC patients. The risk score was higher in patients older than or equal to 65 years than those under 65 in GC patients (Figure 3(a)). There was no difference between GC gender subgroups (Figure 3(b)). The risk score was higher in the G3 group than that in the G1-2 group for the histological grade (Figure 3(c)). Regarding clinical TNM staging, there was no statistical difference between the risk score of patients with stages I-II and those with stages III-IV (Figure 3(d)). Next, we further analyzed the predictive value of the risk score in different clinical characteristics. In the age group less than or equal to 65 years, the prognosis was worse in the high-risk group, whereas in patients older than 65 years, there was no statistical difference in survival between the high- and low-risk groups (Figure 3(e)). The prognosis was worse in both male and female groups in the high-risk group (Figure 3(f)). There were differences in the prognosis of the high-risk and low-risk groups in the G1-2 group, whereas there was no difference in the prognosis of the G3 group (Figure 3(g)). In terms of clinical staging, survival was worse in the stage I-II and stage III-IV groups in the high-risk group (Figure 3(h)). Furthermore, multivariate analysis showed that the risk score was an independent prognostic factor for GC in the TCGA cohort (Figures 4(a) and 4(b)). To further apply the risk score in clinical prognosis prediction, we constructed the nomogram of GC that included risk score for DRR-related gene, TNM stage, gender, grade, and age. Attractively, the nomogram has accurate predictability in GC patients' 1-, 3-, and 5-year overall survival (Figure 4(c)). At the same time, the calibration diagram is listed in the following: decision curve analysis (DCA) demonstrated that the prognostic nomogram was clinically valuable (Figures 4(d) and 4(e)). In summary, the risk score for DRR-related genes can be used as an effective model for predicting survival outcomes of GC patients.

### 3.4. Relationship between Risk Score for DRR-Related Genes and TMB

**Figure 5:**
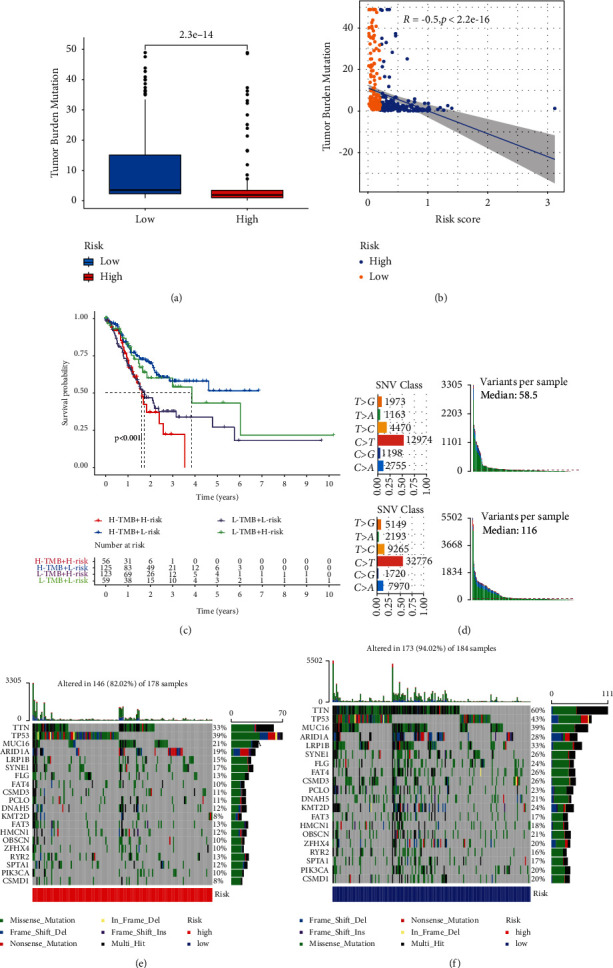
Characteristics of DRR-based risk score in tumor somatic mutation. (a) Difference of TMB between patients from the low-/high-risk subgroups. (b) Correlation between risk score and TMB. (c) Kaplan–Meier curves for patients stratified by both TMB and risk groups. (d) Mutation count per sample in nonsynonymous mutations. (e-f) The waterfall chart was constructed using the low-risk score and high-risk score.

To further elucidate the relationship between TMB and risk score and the effect of both on the prognosis of GC, we first observed that the TMB score was higher in the low-risk group than that in the high-risk group (Figure 5(a), *p* < 0.01) and that the risk score for DRR-related gene was negatively correlated with TMB in GC (Figure 5(b)*R* = −0.5, *p* < 0.01). Next, we further combined the risk score for DRR-related genes and TMB for evaluating the prognosis of GC patients. Interestingly, GC patients with low or high TMB can be further divided into two subgroups based on the risk score for DRR-related genes. Moreover, GC patients with the low-risk score have a superior prognosis than the high-risk score in both low and high TMB subgroups (Figure 5(c), *p* < 0.001). Subsequently, we compared the variation context of the high-risk group and low-risk group, which came from the combination of six variation types (T > G, T > A, T > C, C > T, C > G, and C > A) (Figure 5(d)). The number of each mutation type in the high-risk group was smaller than that in the low-risk group. There was a significant difference in somatic mutation rate among samples. The sweeping landscape of somatic variation shows the various patterns of the top 20 driving genes with the most frequent variation. The significant mutation gene (SMG) landscape showed that the mutation rate of the low-risk group was higher than that of the high-risk group among the top 20 mutation genes (Figures 5(e) and 5(f)). These findings may contribute to a new insight into the relationship between risk scores for DRR-related genes and somatic mutation in GC patients.

### 3.5. Relationship between Risk Score for DRR-Related Gene and TIME in GC

**Figure 6:**
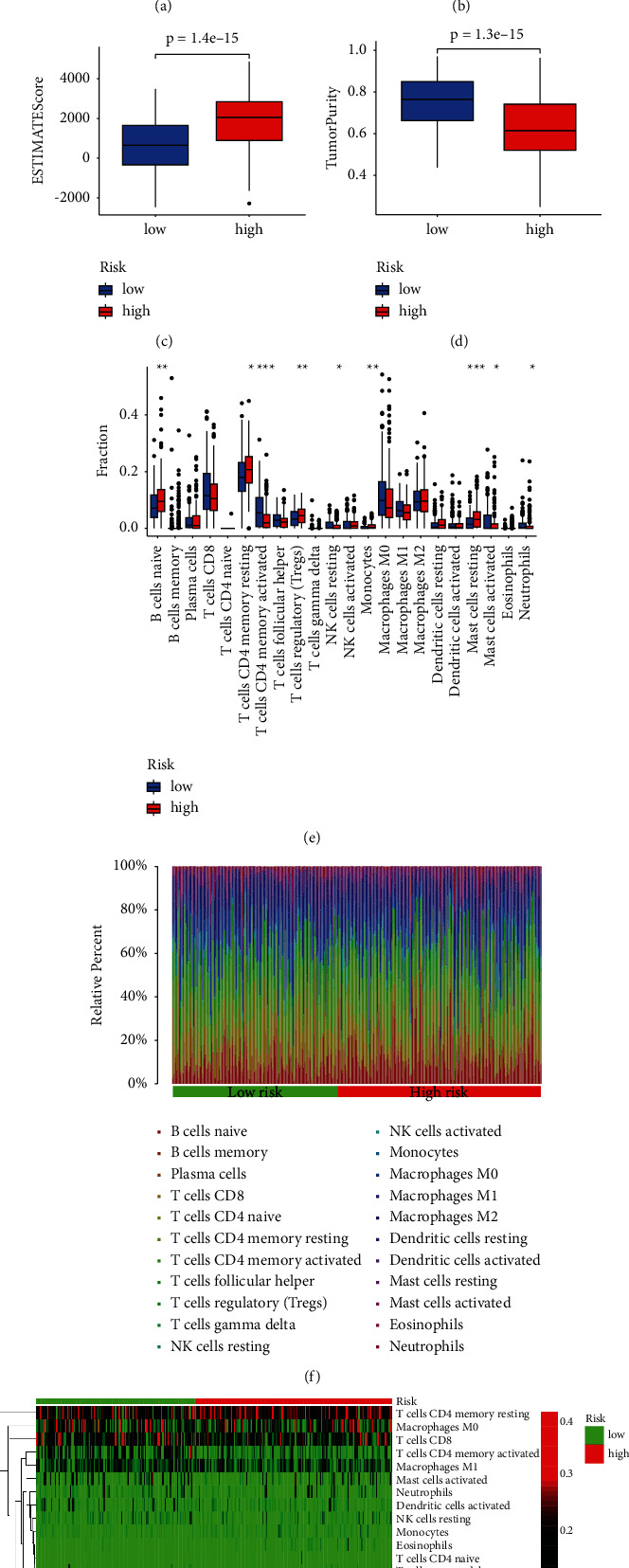
Correlation between prognostic model and immune microenvironment. (a–d) GC patients with high-risk scores have higher ImmuneScore, StromalScore, and ESTIMATEScore than those with low-risk scores. (e) The infiltrating levels of 22 immune cell types in high/low subtypes in the GC. (f) The relative proportion of immune infiltration in high/low-risk groups. (g) The landscape of immune cell infiltration between high/low-risk subtypes. ^*∗*^*p* < 0.05, ^*∗∗*^*p* < 0.01, and ^*∗∗∗*^*p* < 0.001.

The ESTIMATE algorithm was used to score the immune microenvironment of the GC using an “estimation” package to calculate the ImmuneScore, StromalScore, and ESTIMATEScore for each GC patient resulting in four scores: Immunoscore, StromalScore, ESTIMATEScore, and TumorPurity. These four scores were correlated with the risk score for DRR-related genes. The results showed that ImmuneScore, StromalScore, and ESTIMATEScores were higher in high-risk patients and TumorPurity was higher in low-risk patients (Figures 6(a)–6(d), *p* < 0.05). In addition, to determine the relative abundance of tumor-infiltrating immune cells (TIICs) in GC samples, the degree of infiltration of TIICs was estimated using the CIBERSORT algorithm. The immune cell infiltration in the statistically different samples was significantly different in the two groups with higher initial B naïve cells, CD4+ memory resting T cells, Treg cells, monocytes, and mast resting cells in the high-risk patients and more elevated CD4+ memory activated T cells in the low-risk patients (Figure 6(e), *p* < 0.01). The distribution of immune cells in the high- and low-risk groups was also visualized and analyzed (Figures 6(f) and 6(g)).

### 3.6. Prognostic Models with the Correlation between Immune Checkpoints and Immunotherapy of GC

**Figure 7:**
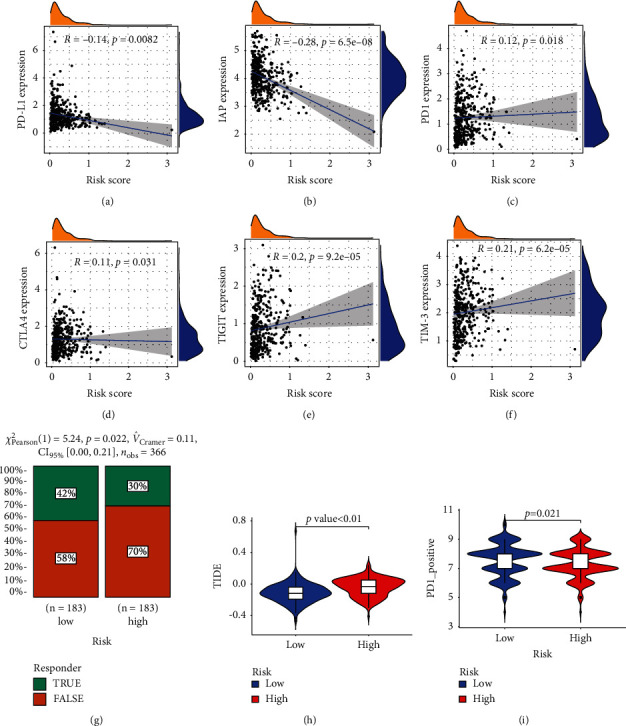
The estimation of two prognostic subtypes in immunotherapy response. (a–f) The expression of six immune checkpoint molecules (PD-L1, IAP, PD1, CTLA4, TIGIT, and TIM-3) in two prognostic subtypes. (g) Chi-squared test plot for immunotherapy in the responder. (h-i) Violin diagram showing the differential TIDE and TCIA between the high/low-risk groups.

Intending to ascertain the efficacy of the risk group for immunotherapy, we initially correlated six common immune checkpoints with the risk score. The results showed that PD-L1 and IAP expressions were negatively correlated with the risk score (Figures 7(a) and 7(b)), R ＜0, *p* < 0.01); however, PD1, CTLA4, TIGIT, and TIM-3 were positively correlated with the risk score (Figures 7(c)–7(f)), R ＞0, *p* < 0.01). Subsequently, the chi-square plot showed that 42% of the responders in the low-risk group were effective, and 58% were ineffective in the TIDE (Tumor Immune Dysfunction and Exclusion); 70% of the responders in the high-risk group were effective, and 30% were ineffective (Figure 7(g), *χ*^2^ = 5.24, *p*=0.022). Ultimately, we evaluated the relationship between risk score and immunotherapy in GC at the TIDE and TCIA (The Cancer Immunome Atlas) (Figures 7(h) and 7(i), *p* < 0.05). Therefore, GC patients with the low-risk score for DRR-related genes showed better immunotherapy outcomes thanin the high-risk group.

## 4. Discussion

DNA is the place where cells store genetic information. The integrity of its structure and function is essential to maintaining life. Therefore, cells evolved specialized DNA repair mechanisms to maintain genome integrity [19, 20]. The significant feature of cancer cells is genomic instability, conducive to the accumulation of mutations and the expansion of tumor heterogeneity [21–23]. DRR mechanism can repair mutant genes in the early stage of the tumor and hinder cancer development. However, DRR-related genes may cause drug resistance of tumor cells to cytotoxic drugs with cancer progression [24]. The occurrence and development of cancer are often accompanied by the inactivation of one or more DRR pathways [25, 26]. Current studies of DNA repair gene prognostic models focused on immediate attachment to DNA repair genes, ignoring the impingement from the TMB [27–29]. Therefore, our research constructed a prognostic model based on a TMB filter of seven DRR-related genes that could better predict the clinicopathological characteristics, survival prognosis, role in the immune microenvironment, and efficacy of immunotherapy in GC patients.

In this study, we have developed a comprehensive description of DRR-related genes based on TMB. This prognostic model may better predict the prognosis and immune microenvironment of individuals with GC, providing a tangible contribution to immunotherapy. In this seven-gene prognostic model, GC patients are divided into a high-risk group and a low-risk group. The prognosis of the high-risk group was worse than that of the low-risk group. The ROC showed that survival at 1, 3, and 5 years had a high prognostic value. Risk curves were assessed and patients' risk increased with increasing scores in the model. Multivariate analysis showed that the prognostic score was an independent prognostic factor. Nomogram showed good prognostic value at 1, 3, and 5 years; calibration chart analysis showed accuracy. There were statistically significant differences between the high-risk and low-risk groups in terms of age and histological grading. At the same time, there were no significant differences in terms of gender and TNM staging. We observed no differences in survival curves in the subgroup survival analysis only for patients with G3 grading. In contrast, the high-risk group had worse prognostic survival than all other subgroups. We further observed habitual differences between prognostic models and tumor mutation profiles. Interestingly, the distribution of TMB was higher in the low-risk group, and the number and frequency of mutations were higher in the low-risk group than those in the high-risk group. The above results suggest that specific mutations in GC may be beneficial for tissue progression. DRR-related genes promote GC progression due to the repair of these mutations.

The high-risk group will have more Treg cell infiltration. It has been reported in the literature that Treg cells allow tumors to produce immune escape by suppressing CD8+ T cells and promoting tumors to express more immunosuppressive molecules [30]. This is consistent with our analysis of immunotherapy. The low-risk group had a lower TIDE and higher TCIA score due to a greater tendency to express PD-L1 and IAP immunosuppressive molecules, suggesting greater effectiveness of immunotherapy in the low-risk group. The findings further elucidate the function of these seven DRR-related genes in GC and may contribute to our understanding of the biology of GC and provide new therapeutic targets. The poor prognosis of GC appears to depend on the multilayered relationship between DNA repair gene mutations, cell proliferation, and immune responses interactions.

LAMA2 is an extracellular protein and is the main component of the basement membrane [31]. It is believed to mediate cell attachment, migration, and tissue during embryonic development through interaction with other extracellular matrix components [32]. Li et al. identified LAMA2 as mediating the activation of the Src family of tyrosine kinase LCK-nondependent T cells by staphylococcal enterotoxin E [33]. Zhang et al. firmly established *LAMA2* as an immune-related gene associated with poor prognosis in pancreatic adenocarcinoma [34].

CREB3L3 encodes members of the alkaline leucine zipper family and the AMP-dependent transcription factor family. The encoded protein is located in the endoplasmic reticulum and acts as a transcription factor activated by cyclic AMP stimulation [35]. Resende et al. found that IL1*β* promoted the transition from chronic gastritis to GC through a CREB-C/EBP*β*-related mechanism [36]. In the meantime, Luan B et al. reported that targeted disruption of CREB or cAMP-regulated transcriptional coactivators 2 and 3 (CRTC2/3) in macrophages downregulated M2 marker gene expression and promoted insulin resistance and facilitated insulin resistance, demonstrating that CREB-related molecules could initiate the human innate immune system [37].

SELP is stored in the alpha granules of platelets and Weibel–Palade vesicles of endothelial cells [38]. This protein redistributes to the plasma membrane during platelet activation and degranulation and mediates the interaction of activated endothelial cells or platelets with leukocytes [39]. Dai et al. screened the TCGA database and found that SELP was highly expressed in GC and significantly correlated with prognosis [40]. Singel et al. analyzed ascites from patients with advanced epithelial ovarian cancer (EOC) and identified that SELP activated neutrophil and platelet responses, promoted metastasis, and hindered antitumor immunity [41].

ABCC9 is a member of the ATP-binding cassette (ABC) transport protein superfamily, transporting various molecules through the outer and inner cell membranes. This protein is thought to form ATP-sensitive potassium channels in cardiac, skeletal, vascular, and nonvascular smooth muscle [42]. Mao et al. reported that ABCC9 was highly expressed in GC and negatively correlated with prognosis, which could be a potential biomarker for GC [43].

CYP1B1 encodes a member of the cytochrome P450 enzyme superfamily. Cytochrome P450 proteins are monooxygenases that catalyze many reactions involving drug metabolism and the synthesis of cholesterol, steroids, and other lipids [44]. Kwon et al. demonstrated that the oncogenic molecular mechanism of CYP1B1 action is associated with specificity protein one-mediated gene regulation, which induces cancer cell proliferation and migration [45]. D'Uva et al. concluded that CYP1B1 is considered a promising target for tumor chemoprevention in the tumor microenvironment due to the involvement of this oncogene in a positive loop with inflammatory cytokines [46]. Thus, evidence suggests that CYP1B1 may be involved in oncogenic events associated with the immune system.

CDH2 belongs to the calmodulin family and is involved in CNS cell adhesion, asymmetric cell division, and presynaptic/postsynaptic processes. For several cancer cells, including lung cancer, the role of CDH2 in cell migration and invasion has been reported. During epithelial-mesenchymal transition (EMT), tumor cells can transform to a CSC-like phenotype with an increase in CDH2 [47]. Hu et al. found that CDH2 promotes EMT in GC cells through LOXL1 overexpression, leading to peritoneal metastasis [48].

The protein encoded by GAMT is a methyltransferase that uses S-adenosylmethionine as a methyl donor to convert guanidinoacetate to creatine. Defects in this gene have been associated with neurological syndromes and hypotonia, possibly due to creatine deficiency and guanidinoacetate accumulation in affected individuals' brains [49]. Liu et al. have identified GAMT as a biomarker of prognosis in patients with advanced GC treated with docetaxel, cisplatin, and S-1 (DCS) [50]. Chen et al. also have demonstrated that high expression of GAMT, a gene driven by DNA methylation, was remarkably associated with poor prognosis [51].

The criteria explored in this study were based on objective indicators that may be more advantageous for detecting immune checkpoint inhibitors that were commonly used in clinical practice. Therefore, the prognostic model we developed was worthy of further study for its predictive efficacy. However, there were inevitable limitations in this study. Although high immune predictive efficacy was observed in the TCGA's STAD datasets, we could not obtain a GC cohort associated with immunotherapy to validate the utility of this study. Furthermore, the translation of these targets into clinical decision-making remains challenging. The mechanisms involved still need further validation in in vivo and in vitro experiments.

## 5. Conclusions

In conclusion, the signature of DRR-related genes are closely interrelated with the prognosis of GC patients. The model based on these seven genes can predict GC patients' response to immunotherapy in GC. Therefore, DRR-related gene signature based on tumor mutation burden is a novel biomarker for prognostic and immunotherapy response in GC patients.

## Data Availability

Publicly available datasets were used in this study. These data can be found in the Cancer Genome Atlas (TCGA) database.

## References

[1] Siegel R. L., Miller K. D., Jemal A. (2020). Cancer statistics, 2020. *CA: A Cancer Journal for Clinicians*.

[2] Sung H., Ferlay J., Siegel R. L. (2021). Global cancer statistics 2020: GLOBOCAN estimates of incidence and mortality worldwide for 36 cancers in 185 countries. *CA: A Cancer Journal for Clinicians*.

[3] Das M. (2017). Neoadjuvant chemotherapy: survival benefit in gastric cancer. *The Lancet Oncology*.

[4] Al-Batran S.-E., Homann N., Pauligk C. (2017). Effect of neoadjuvant chemotherapy followed by surgical resection on survival in patients with limited metastatic gastric or gastroesophageal junction cancer. *JAMA Oncology*.

[5] Gullo I., Carneiro F., Oliveira C., Almeida G. M. (2018). Heterogeneity in gastric cancer: from pure morphology to molecular classifications. *Pathobiology: Journal of Immunopathology, Molecular and Cellular Biology*.

[6] Samstein R. M., Lee C.-H., Shoushtari A. N. (2019). Tumor mutational load predicts survival after immunotherapy across multiple cancer types. *Nature Genetics*.

[7] De Velasco G., Miao D., Voss M. H. (2016). Tumor mutational load and immune parameters across metastatic renal cell carcinoma risk groups. *Cancer Immunology Research*.

[8] Goodman A. M., Kato S., Bazhenova L. (2017). Tumor mutational burden as an independent predictor of response to immunotherapy in diverse cancers. *Molecular Cancer Therapeutics*.

[9] Hurkmans D. P., Kuipers M. E., Smit J. (2020). Tumor mutational load, CD8+ T cells, expression of PD-L1 and HLA class I to guide immunotherapy decisions in NSCLC patients. *Cancer Immunology, Immunotherapy*.

[10] Wang F., Wei X. L., Wang F. H. (2019). Safety, efficacy and tumor mutational burden as a biomarker of overall survival benefit in chemo-refractory gastric cancer treated with toripalimab, a PD-1 antibody in phase Ib/II clinical trial NCT02915432. *Annals of Oncology*.

[11] Li X., Heyer W.-D. (2008). Homologous recombination in DNA repair and DNA damage tolerance. *Cell Research*.

[12] Bhattacharya S., Srinivasan K., Abdisalaam S. (2017). RAD51 interconnects between DNA replication, DNA repair and immunity. *Nucleic Acids Research*.

[13] Gavande N. S., VanderVere-Carozza P. S., Hinshaw H. D. (2016). DNA repair targeted therapy: the past or future of cancer treatment?. *Pharmacology & Therapeutics*.

[14] Ribeiro-Silva C., Vermeulen W., Lans H. (2019). SWI/SNF: complex complexes in genome stability and cancer. *DNA Repair*.

[15] Ying J., Yang L., Yin J. C. (2021). Additive effects of variants of unknown significance in replication repair-associated DNA polymerase genes on mutational burden and prognosis across diverse cancers. *Journal for ImmunoTherapy of Cancer*.

[16] Yimit A., Adebali O., Sancar A., Jiang Y. (2019). Differential damage and repair of DNA-adducts induced by anti-cancer drug cisplatin across mouse organs. *Nature Communications*.

[17] Yap T. A., Kristeleit R., Michalarea V. (2020). Phase I trial of the PARP inhibitor olaparib and AKT inhibitor capivasertib in patients with BRCA1/2- and non-BRCA1/2-mutant cancers. *Cancer Discovery*.

[18] Lin X., Chen D., Zhang C. (2018). Augmented antitumor activity by olaparib plus AZD1775 in gastric cancer through disrupting DNA damage repair pathways and DNA damage checkpoint. *Journal of Experimental & Clinical Cancer Research*.

[19] Tan D. Q., Li Y., Yang C. (2019). PRMT5 modulates splicing for genome integrity and preserves proteostasis of hematopoietic stem cells. *Cell Reports*.

[20] Travers A., Muskhelishvili G. (2015). DNA structure and function. *FEBS Journal*.

[21] Perkhofer L., Schmitt A., Romero Carrasco M. C. (2017). ATM deficiency generating genomic instability sensitizes pancreatic ductal adenocarcinoma cells to therapy-induced DNA damage. *Cancer Research*.

[22] Tubbs A., Nussenzweig A. (2017). Endogenous DNA damage as a source of genomic instability in cancer. *Cell*.

[23] Böttcher R., Kweldam C. F., Livingstone J. (2018). Cribriform and intraductal prostate cancer are associated with increased genomic instability and distinct genomic alterations. *BMC Cancer*.

[24] Rocha C. R. R., Silva M. M., Quinet A., Cabral-Neto J. B., Menck C. F. M. (2018). DNA repair pathways and cisplatin resistance: an intimate relationship. *Clinics*.

[25] Damia G., Broggini M. (2019). Platinum resistance in ovarian cancer: role of DNA repair. *Cancers*.

[26] Wilson D. L., Beharry A. A., Srivastava A., O’Connor T. R., Kool E. T. (2018). Fluorescence probes for ALKBH2 allow the measurement of DNA alkylation repair and drug resistance responses. *Angewandte Chemie*.

[27] Zhang D., Yang S., Li Y. (2020). Prediction of overall survival among female patients with breast cancer using a prognostic signature based on 8 DNA repair-related genes. *JAMA Network Open*.

[28] Wang X.-q., Xu S.-w., Wang W. (2021). Identification and validation of a novel DNA damage and DNA repair related genes based signature for colon cancer prognosis. *Frontiers in Genetics*.

[29] Zhu W., Zhang Q., Liu M., Yan M., Chu X., Li Y. (2021). Identification of DNA repair-related genes predicting pathogenesis and prognosis for liver cancer. *Cancer Cell International*.

[30] Pei X., Wang X., Li H. (2018). LncRNA SNHG1 regulates the differentiation of Treg cells and affects the immune escape of breast cancer via regulating miR-448/Ido. *International Journal of Biological Macromolecules*.

[31] Barraza-Flores P., Bates C. R., Oliveira-Santos A., Burkin D. J. (2020). Laminin and integrin in LAMA2-Related congenital muscular dystrophy: from disease to therapeutics. *Frontiers in Molecular Neuroscience*.

[32] Ahmad K., Shaikh S., Ahmad S. S., Lee E. J., Choi I. (2020). Cross-talk between extracellular matrix and skeletal muscle: implications for myopathies. *Frontiers in Pharmacology*.

[33] Li Z. (2018). Staphylococcal superantigens use LAMA2 as a coreceptor to activate T cells. *The Journal of Immunology (Baltimore, Md.: 1950)*.

[34] Zhang M., Zeng L., Peng Y., Fan B., Chen P., Liu J. (2021). Immune-related genes LAMA2 and IL1R1 correlate with tumor sites and predict poor survival in pancreatic adenocarcinoma. *Future Oncology*.

[35] Nakagawa Y., Wang Y., Han S.-i. (2021). Enterohepatic transcription factor CREB3L3 protects atherosclerosis via SREBP competitive inhibition. *Cellular and Molecular Gastroenterology and Hepatology*.

[36] Resende C., Regalo G., Durães C. (2016). Interleukin-1B signalling leads to increased survival of gastric carcinoma cells through a CREB-C/EBP*β*-associated mechanism. *Gastric Cancer*.

[37] Luan B., Yoon Y.-S., Le Lay J., Kaestner K. H., Hedrick S., Montminy M. (2015). CREB pathway links PGE2 signaling with macrophage polarization. *Proceedings of the National Academy of Sciences*.

[38] Rondina M. T., Voora D., Simon L. M. (2020). Longitudinal RNA-seq analysis of the repeatability of gene expression and splicing in human platelets identifies a platelet SELP splice QTL. *Circulation Research*.

[39] Zhang Z., Guo Y., Qiu C., Deng G., Guo M. (2017). Protective action of Se-supplement against acute alcoholism is regulated by selenoprotein P (SelP) in the liver. *Biological Trace Element Research*.

[40] Dai J., Li Z.-X., Zhang Y. (2019). Whole genome messenger RNA profiling identifies a novel signature to predict gastric cancer survival. *Clinical and Translational Gastroenterology*.

[41] Singel K. L., Grzankowski K. S., Khan A. N. M. N. H. (2019). Mitochondrial DNA in the tumour microenvironment activates neutrophils and is associated with worse outcomes in patients with advanced epithelial ovarian cancer. *British Journal of Cancer*.

[42] Nelson P. T., Jicha G. A., Wang W.-X. (2015). ABCC9/SUR2 in the brain: implications for hippocampal sclerosis of aging and a potential therapeutic target. *Ageing Research Reviews*.

[43] Mao X., He Z., Zhou F., Huang Y., Zhu G. (2019). Prognostic significance and molecular mechanisms of adenosine triphosphate-binding cassette subfamily C members in gastric cancer. *Medicine*.

[44] Maguire M., Larsen M. C., Vezina C. M. (2020). Cyp1b1 directs Srebp-mediated cholesterol and retinoid synthesis in perinatal liver; Association with retinoic acid activity during fetal development. *PLoS One*.

[45] Kwon Y.-J., Baek H.-S., Ye D.-J., Shin S., Kim D., Chun Y.-J. (2016). CYP1B1 enhances cell proliferation and metastasis through induction of EMT and activation of wnt/*β*-catenin signaling via Sp1 upregulation. *PLoS One*.

[46] D’Uva G., Baci D., Albini A., Noonan D. M. (2018). Cancer chemoprevention revisited: cytochrome P450 family 1B1 as a target in the tumor and the microenvironment. *Cancer Treatment Reviews*.

[47] Zhuang Q.-S., Sun X.-B., Chong Q.-Y. (2021). ARTEMIN promotes oncogenicity and resistance to 5-fluorouracil in colorectal carcinoma by p44/42 MAPK dependent expression of CDH2. *Frontiers in Oncology*.

[48] Hu Q., Masuda T., Kuramitsu S. (2020). Potential association of LOXL1 with peritoneal dissemination in gastric cancer possibly via promotion of EMT. *PLoS One*.

[49] Baker S. A., Gajera C. R., Wawro A. M., Corces M. R., Montine T. J. (2021). GATM and GAMT synthesize creatine locally throughout the mammalian body and within oligodendrocytes of the brain. *Brain Research*.

[50] Liu J.-B., Jian T., Yue C. (2019). Chemo-resistant gastric cancer associated gene expression signature: bioinformatics analysis based on gene expression omnibus. *Anticancer Research*.

[51] Chen Z., Liu B., Yi M., Qiu H., Yuan X. (2020). A prognostic nomogram model based on mRNA expression of DNA methylation-driven genes for gastric cancer. *Frontiers in Oncology*.

